# Behavioral changes induced through adenosine A2A receptor ligands in a rat depression model induced by olfactory bulbectomy

**DOI:** 10.1002/brb3.952

**Published:** 2018-04-17

**Authors:** Karla Margarita Padilla, Andres Quintanar‐Setephano, Fabian López‐Vallejo, Laura Cristina Berumen, Ricardo Miledi, Guadalupe García‐Alcocer

**Affiliations:** ^1^ Facultad de Química Universidad Autónoma de Querétaro Centro Universitario Querétaro México; ^2^ Centro de Ciencias Básicas Departamento de Fisiología y Farmacología Universidad Autónoma de Aguascalientes Aguascalientes México; ^3^ Facultad de Ciencias Departamento de Química Universidad Nacional de Colombia Bogotá Colombia; ^4^ Instituto de Neurobiología Universidad Nacional Autónoma de México Juriquilla Querétaro México

**Keywords:** A2A receptors, adenosine, behavioral tests, major depressive disorder, ZM 241385

## Abstract

**Background:**

Major depressive disorders are characterized by their severity and long‐lasting symptoms, which make such disorders highly disabling illnesses. Unfortunately, 50% of major depressive patients experience relapses, perhaps partly because drug research has been performed only in animal models that screen for antidepressant drugs that appear to only ameliorate acute depression symptoms. The bilateral olfactory bulbectomy (OBX) animal model presents the advantage of mimicking the symptoms of chronic depression by means of brain surgery. Adenosine purinergic receptors A2A (A2AR) have been the target of interest in the field of psychiatric diseases. This study aimed to show which A2A receptor ligands exert antidepressive‐like effects in the OBX rat model.

**Methods:**

Forty Sprague‐Dawley male rats were divided into four groups: control, OBX + vehicle, OBX + ZM 241385, and OBX + adenosine groups. Pharmacological treatment was administered for 14 days, and the rats were examined via the forced swim test (FST), open field test (OFT), and sucrose preference test (SPT).

**Results:**

The OBX + ZM 241385 group exhibited decreased immobility time in the FST, decreased isolation time in the OFT, and reversed anhedonia behavior in the SPT compared to the vehicle group. However, no significant differences for adenosine treatment were found.

**Conclusions:**

ZM 241385 administration (2 mg/kg i.p.) restored behavioral changes associated with OBX‐induced depression.

## 1 INTRODUCTION

Major depressive disorder (MDD) is the most common psychiatric disease, with a prevalence of 16.2% among the world population, and it is the third most significant debilitating public health problem (Mathers & Loncar, 2006). The World Health Organization (WHO) predicts that it will be the top debilitating public health problem affecting the world globally by the year 2030 (Kessler & Bedirhan Ustun, 2008). MDD is distinguished from normal sadness by its severity and duration, where mood is affected for extended periods of time. It is characterized by an emotional state that is not under self‐control without an external factor affecting the emotional state and is often accompanied by the inability of patients to experience pleasure. Patients with this disorder exhibit abnormal brain activity associated with neurochemical changes with a deficit of the monoamine system. Additionally, the abnormalities in neuronal system networks involve emotion and reward processes, which depends on heredity background and the environment (A. P. Association and others, 2013; Villanueva, 2013).

To study MDD, acute and chronic animal models have been established. The first such models, including the forced swim test (FST) and the tail suspension test (TST), were developed as empirical screens for antidepressant drugs. However, chronic animal models must be developed based on brain damage, genetic engineering, or environmental manipulation, which result in impairments of brain areas similar to those present in human patients with MMD (Abelaira, Reus, & Quevedo, 2013). Among the chronic models of depression, the bilateral olfactory bulbectomized model (OBX) attempts to resemble the pathophysiology of depression through olfactory bulb removal. The rat bulbar circuitry forms part of the limbic system, which activates functions such as emotions and memory. Thus, ablation of the olfactory bulbs causes marked degeneration of neurons in the olfactory bulb and in other input structures of the limbic system. Therefore, neuroanatomical dysfunction develops in OBX rats, which causes a reduction in the concentrations of neurotransmitters in the cortical–hippocampal–amygdala circuit, mainly serotonin and noradrenalin (Song & Leonard, 2005). Furthermore, behavior changes induced by OBX are reversed through chronic antidepressant treatment, but not acute treatment, showing the predictive validity of the model (Abelaira et al., 2013).

Several drugs are used to treat depression disorders. Typical antidepressants block the reuptake or breakdown of monoamines, but the most prescribed drugs are selective serotonin reuptake inhibitors (SSRI). However, despite the available antidepressants, approximately 30% of nonresponsive patients and a half of all patients suffer relapses (Brunoni, Fraguas, & Fregni, 2009; Machado‐Vieira et al., 2010). Moreover, most current antidepressants have had limited success as they have been developed to ameliorate acute symptoms based on acute animals models, which result in medication break‐up and, thus, the recurrence of depressive symptoms (Duman & Voleti, 2012; Inada & Inagaki, 2015). Accordingly, new treatments are expected to improve mood disorders and should be studied on appropriate animal models of depression. In this context, adenosine purinergic receptors A2A (A2ARs) have been a target of interest in the context of psychiatric diseases due to their broad distribution at the limbic system and their ability to control synaptic plasticity (Ortiz, Ulrich, Zarate, & Machado‐Vieira, 2015). Furthermore, A2ARs can also control the release of serotonin, glial function, and brain metabolic adaptation (Cunha, Ferré, Vaugeois, & Chen, 2008). It is important to note that A2A antagonism had shown promising neuroprotective and anti‐inflammatory mechanisms in the Parkinson's disease using MPTP mouse models (Frau et al., 2011; Gołembiowska, Dziubina, Kowalska, & Kamińska, 2009). Besides, clinical trials reported a reduction in the Unified Parkinson′s disease Rating Scale score in patients with Parkinson′s disease which ingested caffeine during 3 weeks (Postuma et al., 2012).

It remains debated which A2AR ligands exert antidepressant effects. Previous studies reported that a single dose of adenosine (A2AR agonist) induced depressive behavior in rats (Koos, 2011). Experiments by El Yacoubi and collaborators in 2001 later showed reversed signs of behavioral despair in mice treated with selective A2AR antagonists, for example, SCH 58261 and ZM 241385 (ZM) in addition to genetic inactivation of this receptor (El Yacoubi et al., 2001). It was also reported by Kaster et al. in 2004 that adenosine is the cause of reversing depressive behavior in mice (Kaster et al., 2004). Nevertheless, it must be considered that both studies employed the FST and the TST as short‐term exposures to uncontrollable stress, which are acute models and do not reflect depression pathology like chronic models. Notably, a recent work by Kaster et al. in 2015 (Kaster et al., 2015) showed that caffeine consumption (a nonselective A2AR antagonist) prevents mood and memory dysfunction in a chronic stress model.

Considering the abovementioned works, the focus of this research was the evaluation of the antidepressive effect of the A2AR ligands ZM 241385 and adenosine in an animal model that better represents human depression pathology, similar to the OBX model.

## 2 MATERIALS AND METHODS

### 2.1 Animals

Male Sprague‐Dawley rats (200–250 g) were obtained from the Bioterio of National University of Mexico (UNAM). The rats were housed in plastic cages (42 × 27 × 15 cm), six per cage, at 24 ± 1°C under a 12:12‐hr light cycle (lights on at 6:00 a.m.) with free access to food (rat diet, Purina) and water; bedding was changed twice per week. After surgery, the animals were housed alone in small individual cages due to predatory aggression, which is enhanced by bulbectomy (Mucignat‐Caretta, Bondi, & Caretta, 2006). National guidelines for the care and use of animals were followed, and all efforts were made to minimize animal suffering during all procedures. The experiments were approved by the bioethics committee of the institution (34FCN2014).

### 2.2 Bilateral olfactory bulbectomy (OBX)

At the beginning of the study, the rats were randomly divided into two groups, and a modified method proposed by Primeaux (Primeaux, Barnes, & Bray, 2007) was used for bilateral ablation of the olfactory bulbs. The animals were then anesthetized via intraperitoneal (i.p.) injection of 40 mg/kg sodium pentobarbital. The tops of their skulls were swabbed with an antiseptic solution, and a midline frontal skin incision was then made. After skull exposure, 2‐mm‐diameter burr holes (1 mm to the right and left of the midline) were drilled 6 mm rostral to the bregma suture. Both olfactory bulbs were aspirated in OBX rats with a 2‐mm‐diameter glass pipette tip, and special care was taken to avoid damaging the frontal cortex. The skin above the injury was immediately closed with sutures, and 6,000 U of procaine penicillin G plus benzylpenicillin crystalline (PiSA, Mexico) was administered to each rat (intra muscular [i.m.] injection every 24 hr for 3 days) to prevent infection. *Sham*‐operated controls underwent identical anesthetic and drilling procedures as the OBX animals, but their bulbs were not aspirated. A 14‐day postsurgery period was allowed for recovery from the surgical procedure. The animals were monitored daily during this period, and during the first week, paracetamol (Bayer, Germany; 24 mg/kg) was administered to control pain. At the end of the experiment, the rats were anesthetized with sodium pentobarbital (40 mg/kg i.p) and decapitated. The brains were dissected for confirmation of the successful olfactory bulb removal.

Animals with incomplete removal of the olfactory bulbs were removed from the analysis.

### 2.3 Experimental design and treatments

**Figure 1:**
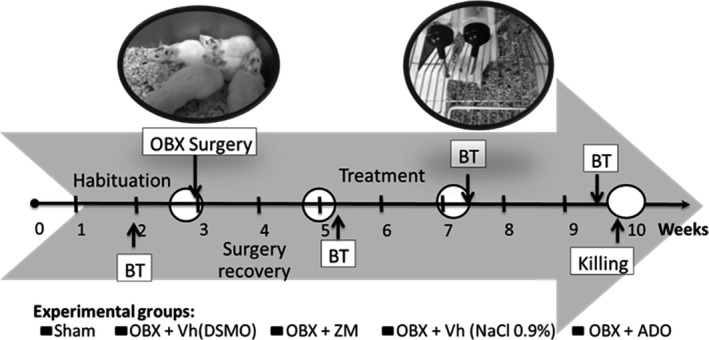
Experimental schedule. Rats were first acclimated to the laboratory conditions for 2 weeks (0–2). At the end of week 2, all animals underwent behavioral tests (BTs). At week 3, the animals were divided into sham surgery bulbectomy (sham) and olfactory bulbectomy (OBX) groups, and the surgeries were performed. OBX group was subdivided into OBX + DMSO (vehicle), OBX + ZM241385 (ZM), OBX + NaCl 0.9%) (Vehicle), and OBX + adenosine (ADO) groups. At week 5, the groups underwent a second round of BTs, and pharmacological treatments were begun (from week 5 to week 10). At weeks 7 and 10, the groups again underwent BTs, and the animals were sacrificed at the end of week 10

The OBX surgery was performed after 2 weeks of habituation. The pharmacological treatment began 2 weeks after recovery from surgery and was maintained for 2 weeks (Figure 1). Abelaira et al. (2013) reported that the pathophysiological changes occurred in depressive patients must also take place in animals, which works as criteria for validity of animal model of depression. Furthermore, the face validity criteria include behavioral manifestation in the animals that mimic the symptoms observed in patients with depression (Abelaira et al., 2013). For OBX model, construct validity is reflected in the following parameters: weigh and hippocampal volume reduction as well as an increase in proinflammatory cytokines. In this study, body weight and anhedonia symptom using sucrose preference test (SPT) was assessed every 2 weeks as a marker of the progression of the OBX‐evoked phenotype. Behavioral tests were conducted before the surgery, after the surgery and post‐treatment (all rats were simultaneously subjected to one test daily, always in the same sequence). After the surgery recovery, the OBX rats were divided into the following four treatment groups (n = 10^−2^): OBX + Vehicle (DMSO), OBX + Vehicle (NaCl 0.9%), OBX + ZM (2 mg/kg), and OBX + (Adenosine 5 mg/kg).

The treatments were administered via i.p. injection at the rate of a single dose per day. ZM 241385 (Sigma‐Aldrich, United Kingdom Lot #024M4712V) was dissolved in DMSO (Sigma Chemical Co., St Louis, U.S.A) and then diluted in chromophore EL (Sigma Chemical Co., St Louis, U.S.A) and 0.9 NaCl % (final concentration: 15%, DMSO and 15% Chromophore EL) according to the manufacturer's instructions. Adenosine (Sigma‐Aldrich, Co., St Louis, U.S.A Lot # SLBGG2403V) was dissolved in 0.9% NaCl. The dosage concentrations were determined based on previous studies (Frau et al., 2011; Gołembiowska et al., 2009; Kaster et al., 2004).

### 2.4 Sucrose preference test

Sucrose preference was measured according to a modification of the protocol reported by Rinwa et al. (Rinwa, Kumar, & Garg, 2013) Briefly, the rats were trained to adapt to a 1% sucrose solution (w/v) for 48 h at the beginning of the experiment, after the training session, the rats were deprived of water and food for 18 hr, followed by the sucrose preference test, in which the rats were housed in individual cages for 4 h and had free access to two bottles that contained 1% sucrose or tap water. To prevent a preference for the position, the location of both bottles was changed every 2 hr during the test. At the end of 4 hr, the sucrose preference (SP) score was expressed as the percentage of the total liquid.

### 2.5 Forced swim test

The forced swim test (FST) was conducted under standard conditions as Castagné et al. (2011) described previously (Castagné, Moser, Roux, & Porsolt, 2011). The rats were placed in a Plexiglass cylindrical tank (45 cm high × 35 cm wide) filled with water at 25°C (water depth: 30 cm). The rats were placed individually into glass cylinders and tested for a six‐min period, of which 2 min are for habituation period and last 4 minutes consisting in the test itself (Abelaira et al., 2013). The test consists in the observation of two behaviors: immobility and struggling. The Immobility was identified when the rat made only the minimum necessary movement to keep its body floating, whereas the struggling behavior was noted when the front paws broke water surface as described by Morales‐Medina et al. (2013) (Morales‐Medina et al., 2013). The latency of immobility and struggling was measured using Etholog 2.5 software. The test was performed before OBX surgery and after OBX surgery to assess the OBX induced‐phenotype. Then, a third test was carried out to test behavioral profile after drug treatment, between the test there was a period of 4 weeks as Babinska and Ruda‐Kucerova (2017) reported (Babinska & Ruda‐kucerova, 2017). All forced swim tests were performed between 10 a.m. and 1 p.m.

### 2.6 Open field test

Locomotor and exploratory behaviors were monitored using an open‐field apparatus consisting of a white wooden box with 40 × 60 × 50 cm dimensions; the opened box was divided into 12 equal squares. The open field tests (OFT) were carried out according to previous studies (Rinwa et al., 2013). Briefly, in a sound‐free room with a 60‐W bulb positioned 1 m from the arena and during the first hours of the active (dark) phase, the rats were placed individually in the center square. Each rat was left to explore the apparatus freely for 10 min, and afterward, the apparatus was cleaned thoroughly with a 10% alcohol solution and dried. During the 10‐min test period, the latency of isolation was measured using Etholog 2.5 software. Isolation was considered when animal remains in only one square of the OFT. Additionally, the number of rat sniffs, grooming, and times when the rats crossed the center was also counted and registered in Etholog 2.5 software.

### 2.7 Analysis

All data are presented as the mean ± SEM. Two‐way ANOVA with repeated‐measures was used to examine whether there was any significant difference for time (presurgery, postsurgery, and post‐treatment) and group [*Sham*, OBX + Vehicle (DMSO), OBX + Vehicle (NaCl 0.9%), OBX + ZM and OBX + ADO]. Additionally, a post hoc Bonferroni's multiple comparison was performed. The *p*‐values from the post hoc comparisons are indicated in the figures. All the statistical analyses were performed using Prisma 5.

## 3 RESULTS

### 3.1 Effects of treatment on body weight

**Figure 2:**
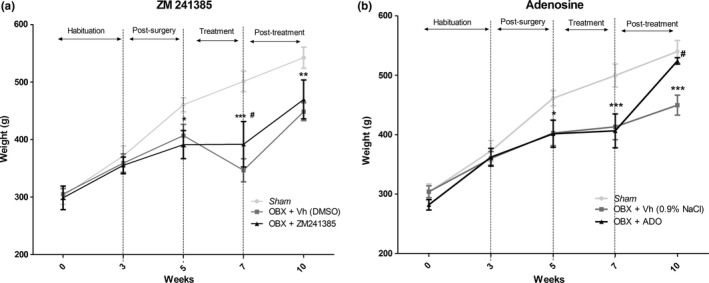
Effects of ZM 241385 and adenosine on body weight. All treatments began 2 weeks after the OBX surgery and were continued for 2 weeks. Each point represents the mean ± SD. Two‐way ANOVA and post hoc Bonferroni's test were used to evaluate differences between groups each week (*p < .05, **p < .01, ***p < .001 indicate significant differences compared to the sham group) (#*p* < .01 indicates significant differences compared to the vehicle group)

Weight was assessed every 2 weeks (Figure 2). There was no significant difference in the body weights among groups during the first 3 weeks. However, on week 5 (after recovery from surgery), a significant decrease in body weight was observed between the OBX groups compared to the *sham* group (*t* = 2.79, *p* < .05). Weight recovery was not detected for the ZM‐treated group after chronic ZM administration (14 days, week 7) compared to the *sham* group (*t* = 19.42, *p* < .001); however, this ZM group also showed a significant weight difference compared to the OBX + vehicle (DMSO) group (*t* = 8.04, *p* < .001), for which the body weight decreased more in the OBX + vehicle (DMSO) group than in the OBX + ZM group. At week 10, all experimental groups had significantly higher body weights than a week 7, but in addition, the *sham* group was again significantly different from the OBX groups (*t* = 12.89, *p* < .001). However, no significant difference was found between the OBX + the ZM group and the OBX + vehicle (DMSO) (*t* = 1.78, *p* > .05) (Figure 2a). The chronic adenosine administration group (OBX + ADO group) did not exhibit weight recovery after 2 weeks of treatment (week 7) compared to the *sham* group (*t* = 3.323, *p* < .01). Nevertheless, on week 10, this group exhibited significant body weight recovery compared to *sham* group (*t* = 2.214, *p* > .05) and a significant difference in weight compared to the OBX + vehicle (NaCl) group (*t* = 14.52, *p* < .001) (*t* = 3.323, *p* < .01) (Figure 2b).

### 3.2 Behavioral effects of OBX and pharmacological treatments

#### 3.2.1 Antidepressant‐like effect of chronic ZM 241385 in the forced swimming test

**Figure 3:**
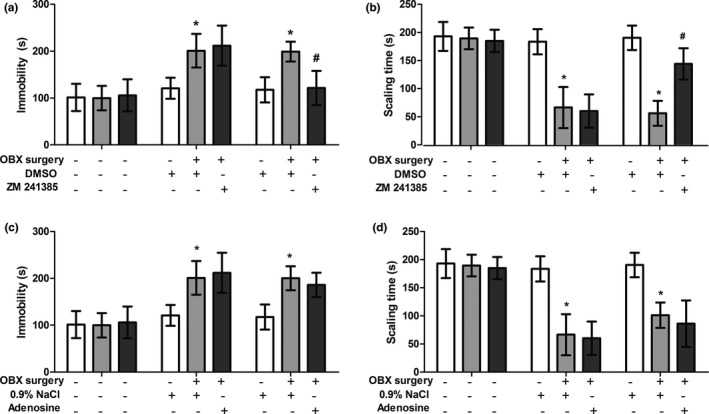
Effects of ZM 241385 and adenosine on the FST. Immobility time in the FST increased significantly after the OBX surgery. (a) The immobility time decreased after administration of ZM 241385 (2 mg/kg, i.p.), and (b) the struggling time increased. (c) Immobility time and (d) struggling time did not recover after adenosine administration. The bars represent the mean ± *SD* (*n* = 10) Two‐way ANOVA and post hoc Bonferroni's test were used to evaluate differences between groups (*p < .05, **p < .01, ***p < .001 indicate significant differences compared to the sham group)

In the FST, immobility time increased by 65% (*t* = 9.85, *p* < .001) after the OBX surgery. Remarkably, the immobility time was reduced by 39% in the chronic ZM group compared to the OBX + vehicle (DMSO) group (*t* = 8.43, *p* < .001) and was not significantly different from the *sham* group (*t* = 0.43, *p* > .05) (Figure 3a). In addition, chronic adenosine administration for 14 days only reversed the increase in the immobility time by 8.5%, and the weight of this group was not significantly different than that of the OBX + vehicle (NaCl) group (*t* = 1.56, *p* > .05) (Figure 3c). Moreover, in the FST, struggling time was also measured; the results showed that struggling time decreased by 65% after OBX surgery (*t* = 16.99, *p* < .001), and neither ZM nor adenosine chronic administration induced a total recovery. Furthermore, both treatment groups (ZM *t* = 3.39, *p* < .05; adenosine *t* = 14.13, *p* < .001) were significantly different than the *sham* group (Figure 3b,d). Although ZM did not reverse the decline of the struggling time compared to the *sham* group, it induced a significant increase in the struggling time by 56% compared to the OBX + vehicle (DMSO) group (*t* = 12.08, *p* < .001), while adenosine had no effect compared to the OBX + vehicle (NaCl) group (*t* = 2.03, *p* > .05).

#### 3.2.2 Effect of chronic ZM 241385 and adenosine treatment in the open field test

**Figure 4:**
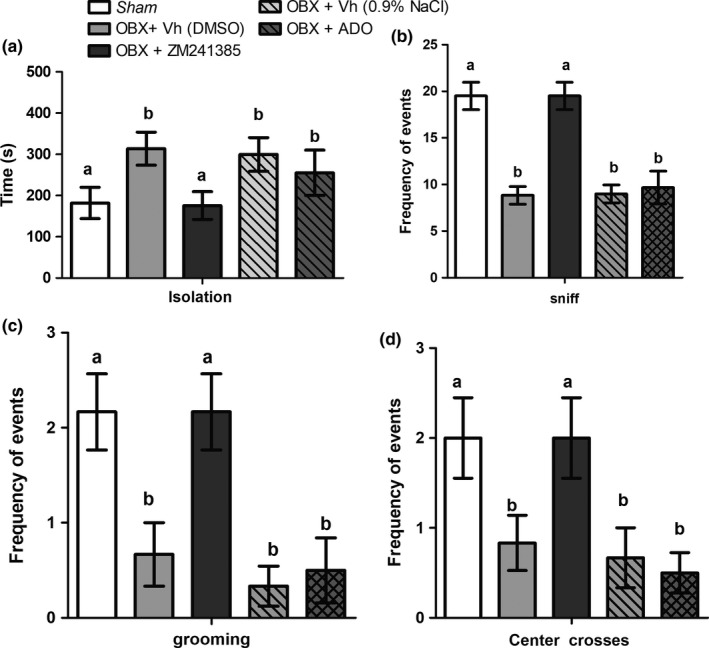
Effects of ZM241385 and adenosine on the OFT in (a) isolation latency, (b) number of sniffs, (c) frequency of grooming and (d) crosses. Frequency of behaviors during 10 min of the OFT. The bars represent the means ± *SD* (*n* = 10^−2^), Two‐way ANOVA was used to evaluate differences between groups (*p < .05, **p < .01, ***p < .001 indicate significant differences compared to the sham group). The different letters (a and b) represent significant differences

To evaluate anxiety behavior, the time that the rats spent motionless in one‐quadrant of the open field was measured as isolation time. The OBX surgery caused a significant increase by 74% in anxiety behavior (*t* = 8.85, *p* < .001) compared to the *sham* group. Figure 4a shows that chronic ZM administration decreased anxiety behavior by 42% (*t* = 9.27, *p* < .001) compared to the OBX + vehicle (DMSO) group. Adenosine treatment decreased anxiety behavior by 10.7% but was not significant (*t* = 1.29, *p* > .05) compared to the OBX + vehicle (NaCl) group (Figure 4b).

The results depicted in Figure 4c show a significant decrease in sniffing as well as in grooming behavior frequencies in the OFT following bulb ablation (*t* = 15.35, ***p < .001 and *t* = 2.68, *p < .05; respectively). Only the ZM treatment exhibited a significant reversal of abnormal behavioral anomalies in OBX rats, corresponding to increased numbers of sniffs (*t* = 15.60, ***p < .001) and grooming (*t* = 2.68, *p < .05) compared to the OBX + vehicle (DMSO) group. Moreover, no significant difference was found between the ZM group and the *sham* group in both behaviors (*t* = 0.45, *p* > .05). Finally, significant changes were found in the number of crossings of the center of the open‐field due to the OBX surgery.

#### 3.2.3 The effect of chronic ZM 241385 and adenosine treatment on anhedonic behavior

**Figure 5:**
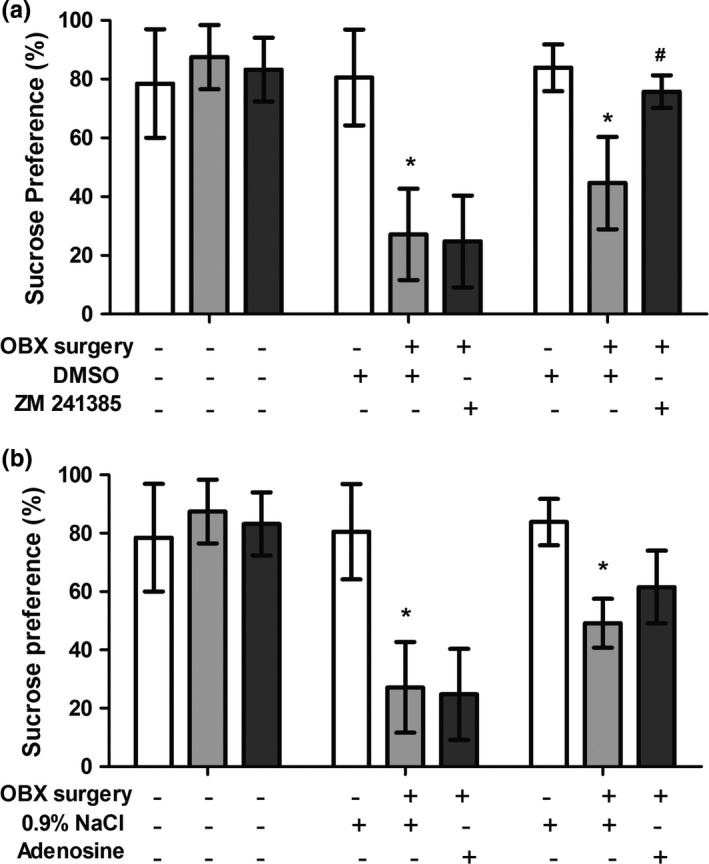
Effects of A2AR ligands on sucrose intake. Sucrose preference significantly decreased after OBX surgery. (a) The A2AR antagonist (2 mg/kg i.p.) restored sucrose preference, whereas (b) A2AR agonist (5 mg/kg i.p.) induced partial recovery, but sucrose preference was not completely restored. The vehicles had no effects on sucrose preference. Each bar represents the mean ± *SD*, and different letters represent significant differences (ANOVA, post hoc Tukey's test)

The sucrose preference test revealed that ablation of olfactory bulbs (OBX) caused a significant reduction in sucrose consumption by 70% compared to the *sham* group (*t* = 15.14, *p* < .001) as shown in Figure 5. This effect was reversed by chronic ZM administration, as the treated group exhibited an increase in sucrose consumption by 69% compared to the *sham* group, and there was no significant difference between the OBX + ZM and *sham* groups (*t* = 2.20, *p* > .05). Chronic adenosine treatment only attenuated sucrose consumption by 25% compared to the OBX + vehicle (NaCl) group (*t* = 3.35, *p* < .01), which was significantly different than the *sham* group (*t* = 6.05, *p* < .001).

## 4 DISCUSSION

To clarify which A2AR ligand exerts the antidepressive effect, animal models must be used that mimic the impaired areas of depression and, additionally, cover the essential criteria of face, content, and construct validity (Abelaira et al., 2013). Thus, antidepressant effects in the OBX animal model were evaluated in this study. Significant behavioral changes in the FST and SPT were found at 2 weeks postsurgery in the OBX group compared to the *sham* group, as previously reported (Hellweg, Zueger, Fink, Hörtnagl, & Gass, 2007; Mucignat‐Caretta et al., 2006). However, the OBX group exhibited significantly decreased ambulation time, although most previous studies reported increased OBX open field activity (Hellweg et al., 2007; Song & Leonard, 2005), this reduction could be associated with fatigue as a residual symptom of MDD (Fava et al., 2014).

The OBX group showed a marked effect of bulbectomy on decreasing body weight (BW), which is consistent with the observed by Hellweg et al. (2007) (Hellweg et al., 2007). Moreover, ZM treatment has not exerted totally weight recovery compared with Sham group, but exerted significant differences in comparison with OBX vehicle. In contrast, adenosine treatment achieves weight recovery of OBX rats; thus, the different results between adenosine and ZM treatment on BW changes could be associated to A1 receptors instead of A2AR as suggest Yang et al. (2015) (Yang et al., 2015). Our results indicated that only chronic administration of the A2AR antagonist ZM reduced the time rats spent motionless and increased struggling behavior. Such effects may be a ZM role in both noradrenergic and serotonergic neurotransmissions associated with the FST behaviors (Castagné et al., 2011), perhaps as recovery of noradrenaline and serotonin neurotransmitter dysfunction caused by the OBX model (Song & Leonard, 2005).

Furthermore, chronic ZM treatment exerted an anxiolytic effect, increasing ambulation time. This finding can be explained by the attenuation of basolateral amygdala (BLA) hyperexcitability, which has been proven to play a key role in anxiety disorder manifestations (Mahan & Ressler, 2012; Rau, Ariwodola, & Weiner, 2015). In contrast, a previous work reported that A2AR striatum selective depletion induces anxiety‐like symptoms in rats (Wei et al., 2014); however, it has been shown that anxiety‐like behavior depends on the brain region where A2ARs are depleted (Yamada, Kobayashi, & Kanda, 2014). Additionally, chronic ZM administration improved grooming behavior frequency in the OFT, which is consistent with 2‐week daily administration of imipramine in OBX rats (Rinwa et al., 2013).

The results obtained in this study are consistent with those reported for A2AR antagonists that reverted the anhedonia‐like (loss of interest or pleasure) condition in OBX rats, which is the main depression diagnostic criteria (DSM‐V) and is consistent with previous studies (Kaster et al., 2015) and escitalopram treatment (Kurhe, Mahesh, Gupta, & Devadoss, 2014). The significant increase in sucrose preference of the OBX + ZM group may be due to the fact that blocking A2ARs could improve motivational dysfunction through regulation of the mesolimbic dopaminergic circuit involved in effort‐related decision making (Yohn et al., 2015). Moreover, ventral tegmental area (VTA) dopaminergic neurons play an important role in depression‐like reward behaviors (Tye, Miller, & Blaha, 2013). Additionally, A2ARs can form heteroreceptors with D2 and D3 dopamine receptors on striatal neurons and in the nucleus accumbens (Marcellino et al., 2007). Therefore, ZM may exert its anti‐anhedonia effect by regulating dopaminergic signaling in reward neuronal circuits. Taken together, the present findings provide evidence of behavioral changes, such as effects on neuronal circuits involved in depression‐like behaviors, associated with antidepressive effects of ZM. Remarkably, there are homologous neuronal circuits in animals and humans (Keeler & Robbins, 2011).

In summary, we found significant behavioral changes with chronic ZM administration of 2.0 mg/kg i.p. per day for 2 weeks, whereas we did not observe such effects with daily adenosine treatment for 2 weeks (5 mg/kg i.p.). In this context, previous studies showed that one single dose of ZM (15 mg/kg i.p.) exerts antidepressive‐like effects in the FST model (El Yacoubi et al., 2001), whereas Kaster et al. (2004) reported different results for the effects of adenosine administration (single dose of 5 mg/kg i.p.). The difference between the results obtained in acute and/or chronic animal models may be because, in the FST and TST, only a single dose of drug was administered before the tests, while the behavioral changes induced by the OBX model can only be reversed by chronic antidepressive treatments but not acute treatments (Abelaira et al., 2013). However, previous studies have been reported in which clinical antidepressant action requires chronic administration to obtain favorable results (Castagné et al., 2011; Rinwa et al., 2013). Otherwise, antidepressant effects of the administration of differential acute (single administration) or chronic drug doses have been reported. For example, in a previous study, an effective 5 mg/kg p.o. acute dose of KW‐6002 (selective A2AR antagonist) and effective 1.25 mg/kg p.o. chronic doses were reported (Yamada, Kobayashi, Mori, Jenner, & Kanda, 2013).

## 5 CONCLUSION

We conclude that 2 weeks of ZM administration (2.0 mg/kg i.p.) was more effective than adenosine treatment (5 mg/kg i.p.). ZM reversed the effect of the OBX on behavioral deficits associated with depression‐like behavior. Thus, ZM induced antidepressive‐like effects, including recovery of behavioral despair, anxiety, and anhedonia symptoms. Nevertheless, to elucidate its detailed mechanism in a chronic depression model, further studies are required.

## CONFLICTS OF INTEREST

None declared.
